# Environmental Fate of Organic Sunscreens during Water Disinfection Processes: The Formation of Degradation By-Products and Their Toxicological Profiles

**DOI:** 10.3390/molecules27144467

**Published:** 2022-07-13

**Authors:** Antonio Medici, Giovanni Luongo, Giovanni Di Fabio, Armando Zarrelli

**Affiliations:** Department of Chemical Sciences, University of Naples Federico II, Via Cintia 4, I-80126 Naples, Italy; antonio.medici@unina.it (A.M.); giovanni.luongo@unina.it (G.L.); difabio@unina.it (G.D.F.)

**Keywords:** sunscreens, chlorination, hypochlorite, degradation byproducts, water treatment, acute toxicity

## Abstract

The development of any commercial product should also be aimed at reducing the risk associated with it, according to the safe-by-design concept; that is, risk assessment should always be at the center of the design, and the impact on human and environmental health should be assessed and eliminated during the product development phase and not afterwards. Unfortunately, even today, most operators in any production sector implement the philosophy of “risk management” or rather of managing the problem when it occurs, using spot interventions instead of changing the approach. This argument is also valid in the production of solar filters, which have reached a satisfactory degree of efficiency in the face of a substantial underestimation of the risks associated with their possible environmental fate. In fact, solar filters have been found in bathing waters and their environmental fate may depend on various factors such as the pH of the water, the presence of organic material, metal ions and light, and, above all, the chemical agents used in the disinfection of the water itself. Thus, during disinfection processes, the generation of dozens of products with a lower molecular weight and generally of an aromatic nature has been tested, where some of them did not receive an exact structural definition and a precise evaluation of their precise toxicological profile. Therefore, it is interesting to draw a complete picture of organic sunscreens and of the byproducts obtained under different conditions and their related ecotoxicological profile.

## 1. Introduction

A sunscreen is a product that can reflect or transform the sun’s rays, protecting skin structures from potential damage induced by ultraviolet UVA and/or UVB radiation. Keeping with this definition, sunscreens are substances that, when applied to the skin through cosmetic formulations, interfere with UV rays, reducing their deleterious effect on the health of the skin itself. Humans have always tried to protect their skin from the sun’s rays, first with skins and clothing, then with accessories of a different nature that are still used in the present day. The first use of sunscreens dates to the end of the nineteenth century, when Friedrich Hammer published a monograph on the effectiveness of different chemicals in protecting against sunburn. From here, great steps were taken in a short time, facilitating the marketing of the first solar filter in the United States in 1928. These events opened the doors to experimentation in the cosmetic and dermatological fields, promoting the identification of numerous compounds in the following years that could guarantee an effective shielding action against UV rays. However, only in the early 1990s, with the introduction of inorganic particles, did UV protection reach high levels of effectiveness, offering active protection even for the most sensitive skin. In the coming years, researchers will focus on developing sunscreens that are environmentally friendly and free from potential dangers to human health. The heterogeneous category of sunscreens includes chemical compounds that are capable of interacting with incident UV rays and causing reflection, dispersion, or absorption phenomena. Based on their chemical nature and mechanism of action, sunscreens are classically divided into physical (or inorganic) and chemical (or organic) filters. In particular, the former is mostly made up of particles of mineral origin, such as titanium dioxide; these sunscreens are effective in forming a surface that reflects the UV rays incident on the skin and absorbs and converts the radiation into a less harmful form. These molecules can be further divided into UVA, UVB, or complete filters, depending on their protection spectrum. Unlike chemical sunscreens, they do not retain heat or penetrate the skin. Furthermore, they are not broken down, do not interact, and are not altered or damaged by solar radiation. These are inert, photostable substances, particularly safe for the health of the skin. The chemical (or organic) filters consist of molecules that are capable of permeating the superficial skin layer, absorbing part of the UV radiation, and dissipating the energy in the form of wavelengths that are not harmful to the skin. Chemical filters offer a greater selection in terms of the quality of radiation that reaches the skin, allowing it to be protected while allowing (at least partially) tanning.

**Figure 1:**
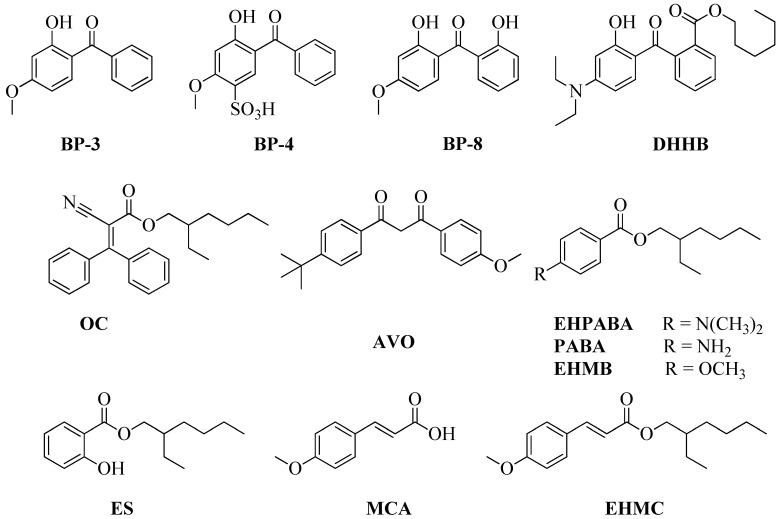
Structures of the most common commercial organic sunscreens.

The major difference between physical filters and chemical filters lies in the fact that the former allow for the attenuation of radiation across the entire UV spectrum, while the latter must be combined with each other to obtain complete (broad spectrum) protection. This synergy of action, if well calibrated, allows manufacturers to obtain products with better performance. Chemical or organic filters are a heterogeneous group of organic molecules (Figure 1, Table 1 and Table 2) which, due to the effect of photochemical excitation, can modify their structure, resulting in a loss of activity. In some cases, the formation of degradation products has been shown to cause potential local and systemic toxicity, up to the involvement of the immune and endocrine systems [1,2,3,4].

Some chemical filters, such as PABA and some of its derivatives, have been banned in the European Union and in other countries around the world. The problems associated with protective lotions are related not only to the interaction of their organic filter with solar radiation but also specifically to the contact of the organic filter with water, depending on its pH and composition, or with agents that are used for the disinfection of the water itself.

Apparently, sunscreens, even those that are generally considered safe, can form more dangerous substances if they meet chlorine. Thus, it is important to ask which sunscreens react and why as well as whether complete ecotoxicological profiles have been drawn up. Nowadays, UV filters are considered as emerging contaminants, having been detected in swimming pool water [8,19]; wastewater [20,21]; the surface waters of rivers [22,23], lakes [24], and seas [22,23]; groundwater [25]; drinking water [26,27]; sediments [28]; and even in animals [21]. Unfortunately, there is generally very little data available on the environmental and human health risks associated with their presence. Several studies have recently been published that identify how sunscreens can interact with all forms of life. The chemicals used in sunscreens are very potent hormones and as such are classified as endocrine disruptors. Released into the body, they can act as estrogen, anti-estrogen, testosterone, or anti-testosterone, disturbing the natural hormonal balance. For example, by administering sunscreen components such as **BP-3** and **EHMC** to rats, the researchers found that the weight of the rats’ uteruses increased, which indicates that these substances exert an estrogenic action. The use of sunscreens has been so hyped in the USA that **BP-3** is now found in the blood of 97% of Americans, including the 90% who say they have never used sunscreens. This substance has such widespread use that it has managed to invade our aqueducts, and phyto-purification plants are unable to eradicate it. Since it is ubiquitous in many products, it now circulates in the bloodstream of many people whether they use sunscreens or not [29,30,31].

In the literature there are many articles on the chemical nature of sunscreens, their mechanisms of action, and their degree of efficiency, but there are none that consider their possible environmental fate during the disinfection processes of water, in which they are found in ever greater concentrations. Therefore, the purpose of this work was to trace a complete picture of the byproducts obtained via water disinfection processes and their related ecotoxicological profiles for the most important organic sunscreens from a commercial point of view.

## 2. Discussion

In Europe, Annex VI of EC regulation no.1223/2009 (30 November 2009) and its subsequent amendments established that the chemical agents approved for use in sunscreens and cosmetic products are different and can be divided into UVA chemical filters, e.g., derivatives of benzophenone (**BP-3**, **BP-4**, **BP-8**, **DHHB**, **OC**) and dibenzoylmethane (**AVO**), and UVB chemical filters, including derivatives of *p*-aminobenzoic acid (**EHPABA**, **PABA**, **EHMB**), salicylic acid (**ES**), and cinnamic acid (**MCA**, **EHMC**).

Regardless of the molecule taken into consideration, a good solar filter—to be considered as such—should possess the following requisites: a wide absorption spectrum (280–380 nm); good chemical stability and good photostability; a high extinction coefficient; good solubility, compatibility and stability in the finished product (including packaging); a good toxicological profile (i.e., very low acute and long-term toxicity, absence of phototoxicity, non-sensitizing, non-photosensitive, absence of percutaneous absorption, etc.); good surface action (i.e., non-absorbable, non-irritating, and well tolerated by skin and mucous membranes); an inability to cause the discoloration of skin and fabrics; and low or no odor. The most commercially important products and their possible derivatives from the chlorination reaction of the waters in which they are found are listed below.

### 2.1. UVA Chemical Filters: Benzophenone Derivatives

**Scheme 1:**
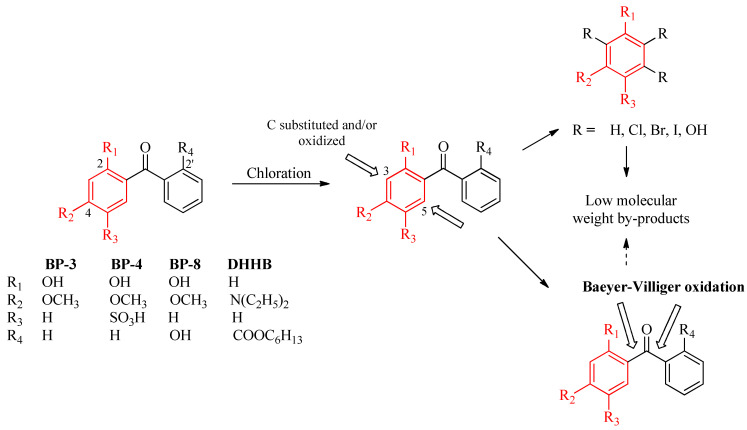
Chemical filters derived from benzophenones and possible reactive sites.

There are four derivatives of benzophenone, namely **BP-3**, **BP-4**, **BP-8,** and **DHHB**. All have an electron donor group on the C-4 carbon with respect to the bond with the carbonyl, and three of these (**BP-3**, **BP-4,** and **BP-8**) have a hydroxyl group on the C-2 carbon (Scheme 1); this makes one of the two aromatic rings (shown in red in Scheme 1) more easily activated to undergo electrophilic substitution reactions.

In fact, in pure, drinkable, or sea water at different pH levels, the chlorination reaction involves the formation of variously substituted derivatives in the C-3 and C-5 positions. These products can then undergo subsequent degradation with the breaking of the C-1–CO bond and the formation of the corresponding C_6_C_0_ skeletal aromatic compounds. The latter then lead to the formation of a series of halogenated/hydroxylated derivatives of methane, ethanes, and the corresponding acetic acids. The possibility of obtaining both Baeyer–Villiger oxidation products is noteworthy, in turn possible precursors of the previous the C_6_C_0_ aromatic compounds.

Below, a detailed analysis of the oxidation products obtained from each benzophenone derivative is given.

#### 2.1.1. 2-Benzoyl-5-methoxyphenol (**BP-3**)

**Scheme 2:**
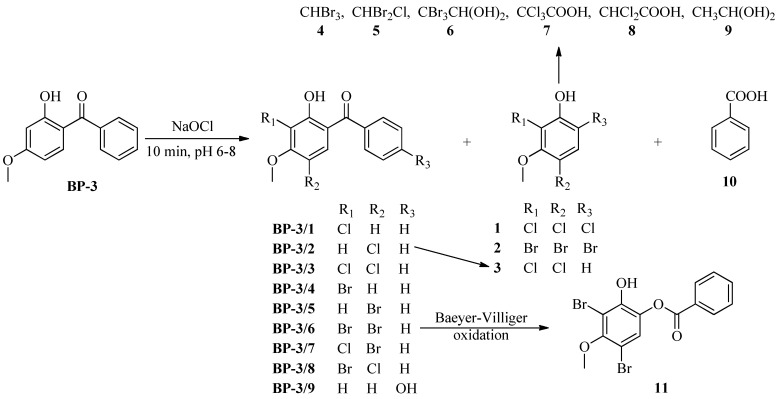
Disinfection by-products from **BP-3** chlorination.

At concentrations between 10 and 50 ng/mL and by treatment with sodium hypochlorite at concentrations between 0.1 and 3.0 μg/mL, at a pH between 6 and 8, and at a temperature of about 20 °C, **BP-3** degrades almost completely (Scheme 2) in about 10 min, with pseudo-first-order degradation kinetics [32]. In particular, **BP-3** has a half-life time (t_1/2_) of 2–3 min in pure water and in the absence of bromide ions; however, in drinking water and in the presence of bromide ions, the t_1/2_ is at least halved.

**BP-3/1-8** products can be obtained in both pure and potable water, using either the pure standard or by chlorinating commercial solar filters. The di- or tri-halogenated phenols (**1-3**) resist subsequent degradation [32].

Manasfi et al. [33], when investigating the oxidation of **BP-3** in artificial seawater, found the same **BP-3/1-8** products beyond ester **11**—which was assumed to be derived from a Baeyer–Villiger oxidation reaction of the corresponding ketone **BP-3/6**—and the following oxidation products: bromoform (**4**), dibromochloromethane (**5**), tribromoacetaldehyde hydrate (**6**), trichloroacetic acid (**7**), dichloroacetic acid (**8**), 1,1-ethanediol (**9**), and benzoic acid (**10**).

Under the same conditions, other studies have also shown the presence of 2,4,6-trichloro-3-methoxyphenol (**1**), 2,4-dichloro-3-methoxyphenol (**3**), chloral hydrate, and chloroform [34,35].

**Scheme 3:**
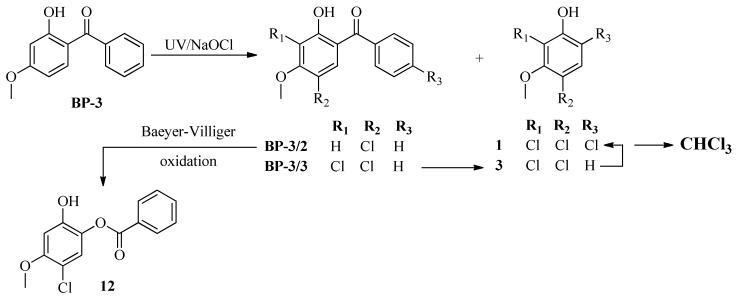
Disinfection by-products from **BP-3** UV/chlorination.

In the UV/chlorination process, it is possible to obtain the oxidation of the C-4 carbon in the unsubstituted aromatic ring to generate the compound **BP-3/9**, product **12**, via the Baeyer–Villiger oxidation of **BP-3/2** and chloroform, the final product obtained starting from **BP-3/3**, through the phenolic byproducts **3** and **1** (Scheme 3) [35].

In these reactions, the reactivity of the OH radical increases as the pH decreases (HOCl reduces OH less than ClO^−^), while the kinetics are promoted by the HCO_3_^−^ ion (the true active species is CO_3_^−^, radical formed by reaction with OH and/or Cl, which is reactive towards electron-rich compounds such as phenols) and inhibited by the presence of humic acids which compete with **BP-3** in reaction with reactive species. It is interesting to note how the reaction mixtures obtained during the simple chlorination process and during chlorination combined with UV treatment are more toxic for short reaction times, as demonstrated by bioluminescence measurements on the *Vibrio fischeri* bacterium. This would indicate the generation of additional toxic products in the first stages of the reactions, such as product **1** [34]. In particular, mixtures obtained with chlorination alone seem more toxic than those obtained with the two combined treatments, indicating either the lower toxicity of compounds **BP-3/9** and **12** or the increased degradation of the starting compound [35].

#### 2.1.2. 5-Benzoyl-4-hydroxy-2-methoxybenzenesulfonic Acid (**PB-4**)

**Scheme 4:**
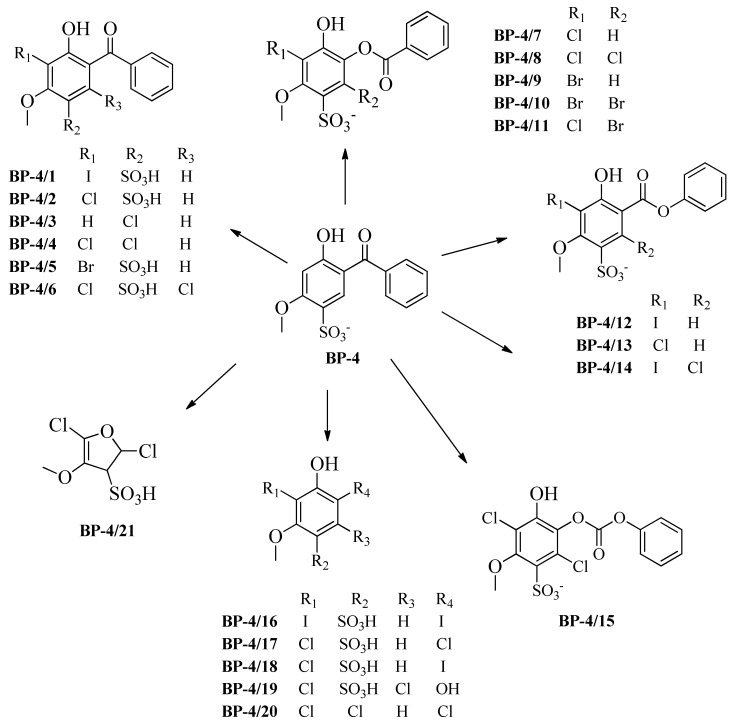
Disinfection by-products from **BP-4** chlorination.

The **BP-4** product was subjected to an oxidation reaction, mimicking a disinfection process with sodium hypochlorite in the presence of an iodide ion. Under these conditions, the iodide is rapidly oxidized to form hypoiodous acid (HIO), obtaining five iodo derivatives, the compounds **BP-4/1**, **12**, **14, 16,** and **18**, at three different pH values (4.6, 7.5, and 11.0), as identified by mass spectrometry studies (Scheme 4) [36].

Seventy-five percent of the starting product degrades in the first 5 min; correspondingly, the **BP-4/1** iodinated product reaches its maximum concentration in about 30 min and then slowly decreases, clearly indicating how this product is the precursor of others formed in the course of the reaction. In general, it should be kept in mind that iodo derivatives are formed in lower quantities than chlorine derivatives, that the monohalogenation of the substrate is favored over double halogenation and, finally, that the Baeyer–Villiger oxidation products are quickly formed and hydrolyzed. Generally, it has been observed that at an acidic (4.6) or neutral (7.5) pH there are higher kinetics and reaction yields for all products compared to a basic pH (11.0). This can be explained considering that at a basic pH, the conjugated bases of HClO and HIO predominate, which are less reactive and mainly determine monohalogenation reactions. On the other hand, iodide ions in the presence of HClO involve: (i) the decrease in the concentration of HClO, which oxidizes the iodide ions to IO_3_^−^ and (ii) the IO_3_^−^ species’ ability to attack the by-products of **BP-4,** giving rise to reactive species that compete with **BP-4** itself in the reaction with active chlorine. All this implies that the degradation of **BP-4** as a function of pH, after 2 h of treatment, goes for no more than 70% at acid pH, 80% at neutral pH, and almost 100% at basic pH [36].

Negreira et al. [37] verified that the half-life time of **BP-4** in pure water decreased from 5.25 to 0.98 min as the concentration of active chlorine and pH (from 6.3 to 8.1) increased; moreover, a dramatic decrease in the half-life times (about 0.2 min) then occurred in the presence of bromide ions. Tests performed to evaluate the ecotoxicological profiles of the mixtures indicated a higher toxicity for those obtained by chlorinating the starting product in the presence of iodine than for those obtained in its absence and a greater toxicity of the mixtures obtained at a basic pH [36].

#### 2.1.3. 4-Methoxy-2,2′-dihydroxy-benzophenone (**PB-8**)

**Scheme 5:**
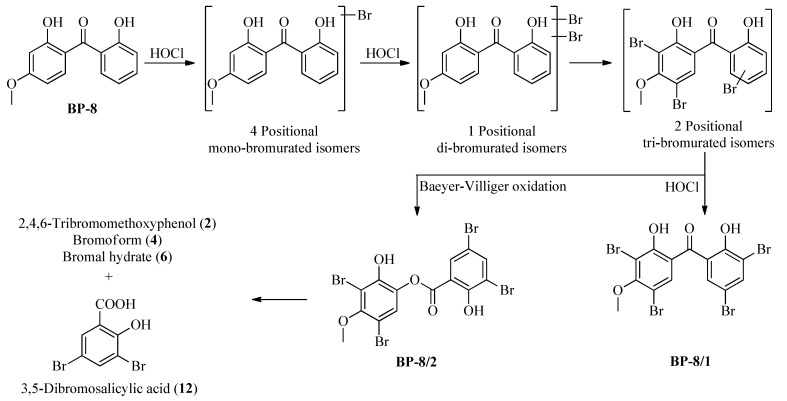
Disinfection by-products from **BP-8** chlorination.

The course of the chlorination reaction of **BP-8** (Scheme 5) in seawater (with a concentration of bromide ions equal to 67 mg/L) is very similar to that of **BP-3**. The reaction kinetics are faster than those in pure or drinking water. Mass studies have revealed the presence of four monobrominated, one dibrominated, two tribrominated, and one tetrabrominated (**BP-8/1**) isomer in addition to the **BP-8/2** ester obtained from a Baeyer–Villiger oxidation reaction. The last two products were the only ones for which the exact structural definition was possible. It is presumed that the degradation products **2**, **4**, **6,** and **12** are obtained from **BP-8/2** [33].

#### 2.1.4. Hexyl 2-(4-diethylamino-2-hydroxybenzoyl)benzoate (**DHHB**)

**Figure 2:**
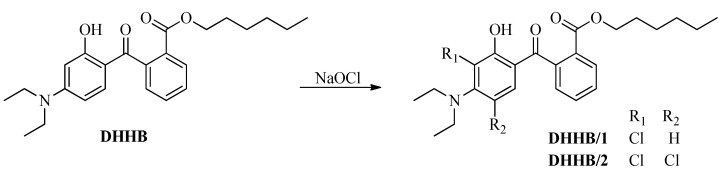
Disinfection by-products from **DHHB** chlorination.

The reaction between pure **DHHB** and sodium hypochlorite at a three-times-higher concentration by weight was considered in [9]. The results showed that the degradation kinetics varied as a function of the pH and temperature of the water and of the concentration of free chlorine but were substantially unaltered in the presence of organic material dissolved in the solution (DOM, 1–3 mg/L). In particular, the degradation rate was maximum at a pH value of 4.0 in the presence of free chlorine equal to three times the quantity by weight of the solar filter and at a temperature of 25 °C, with the formation of volatile by-products with a lower molecular weight. Conversely, chlorination products were present in greater quantities at higher pH (7.5) and lower free chlorine concentrations (equal by weight to the quantity of DHHB present in solution) and at a temperature of 15 °C. Under these conditions in drinking water, the solar filter degraded with a half-life of about 2 min and formed two products, mono- and dichlorinated, as determined by MS analysis (Figure 2).

The latter tend to accumulate when the degradation rate of **DHHB** is low. When exposed to a solar lamp, the filter was also shown to be very stable for photodegradation, with a degradation percentage not exceeding 15% after an exposure of at least 6 h and, in any case, without the formation of detectable by-products.

The **DHHB** compound and its chlorinated derivatives were tested on the luminescent bacterium *Vibrio fischeri*, on the unicellular green alga *Pseudokirchneriella subcapitata,* and on the crustacean *Daphnia magna*. There were no major differences in activity between the two chloro derivatives; specifically, they showed activity comparable to DHHB on the bacterium (EC_50_ 85–86 vs. 0.96), higher on the alga (EC_50_ 0.12–0.18 vs. 0.01), and lower on the crustacean (EC_50_ 0.89–1.03 vs. 1.42) [8].

#### 2.1.5. 2-Ethylhexyl-2-cyano-3,3-diphenylacrylate (**OC**)

**OC** is a solar filter that shows, perhaps most of all, poor reactivity, justified by the lack of electron donor substituents on its aromatic ring; consequently, the compound is not very sensitive to electrophilic attacks, remaining practically unchanged regardless of the dose of chlorine used and the reaction time [38]. In fact, the reaction course was evaluated with UV filter/chlorine ratios of 1:10, 1:25 and 1: 100 and with a chlorine concentration in line with that used in disinfection processes without any apparent result. The same results were also observed in chlorinated seawater pools, where the presence of high concentrations of bromide (up to 70 mg/L) resulted in a higher reactivity of UV filters than in chlorinated freshwater pools. In fact, the tests showed that the OC remained unchanged even at a slightly alkaline pH (about 8) when the added chlorine reacted with the bromide ion forming the HBrO/BrO^−^ pair, which usually reacts with phenolic compounds about 1000 times faster than chlorine, with the concrete possibility of forming bromine derivatives with toxicity that is well known.

### 2.2. UVA Chemical Filters: Dibenzoylmethane Derivatives

**Figure 3:**
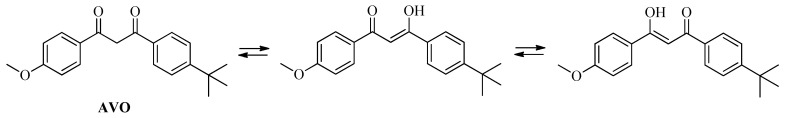
Keto-enol tautomers of **AVO**.

Dibenzoylmethane derivatives constitute an important family of ultraviolet filters that are used in numerous commercial products. The most commonly used is 4-tert-buthyl-4′-methoxydibenzoylmethane (**AVO**), commercially known as avobenzone, Parsol 1789, or Eusolex 9020, from which more than 70 degradation products have been isolated and identified after oxidation processes. In addition, the formation of a large number of derivatives with the same skeleton as the starting product has been observed; these derivatives are variously substituted either on the aromatic rings, differently from each other but both substituted with activating electron donor groups, or at the α carbon between the two carbonyls, likely through the two enol forms shown in Figure 3. Furthermore, a large number of skeletal compounds, including C_6_C_0_, C_6_C_1_ and C_6_C_2_, variously oxidized and substituted, as well as Baeyer–Villiger oxidation products, have been isolated and identified.

#### 4-Tert-butyl-4′-methoxy-dibenzoylmethane (**AVO**)

4-tert-buthyl-4′-methoxydibenzoylmethane exists in two tautomeric forms (Figure 3), of which enol is the predominant form, with a maximum absorption between 350 and 365 nm depending on the solvent used [39].

In one study, the compound was detected in surface waters up to a concentration of 20 ng/L. If irradiated, the enol form tautomerizes to the ketone, which is then photodegraded. Two commercial products were individually dissolved in water and then treated with 4% NaClO in the presence of dissolved organic material, obtaining the identification of two avobenzone derivatives, with one (**AVO/70**) and two chlorine atoms (**AVO/71**), at the alpha carbon between the two carbonyl groups [39]. Obtaining these two by-products shows how the commercial product has its own reactivity and, consequently, can be transformed. It is less soluble in water than the pure product and therefore is evidently more recalcitrant to degradation itself in conditions close to that of a normal swimming pool.

**Scheme 6:**
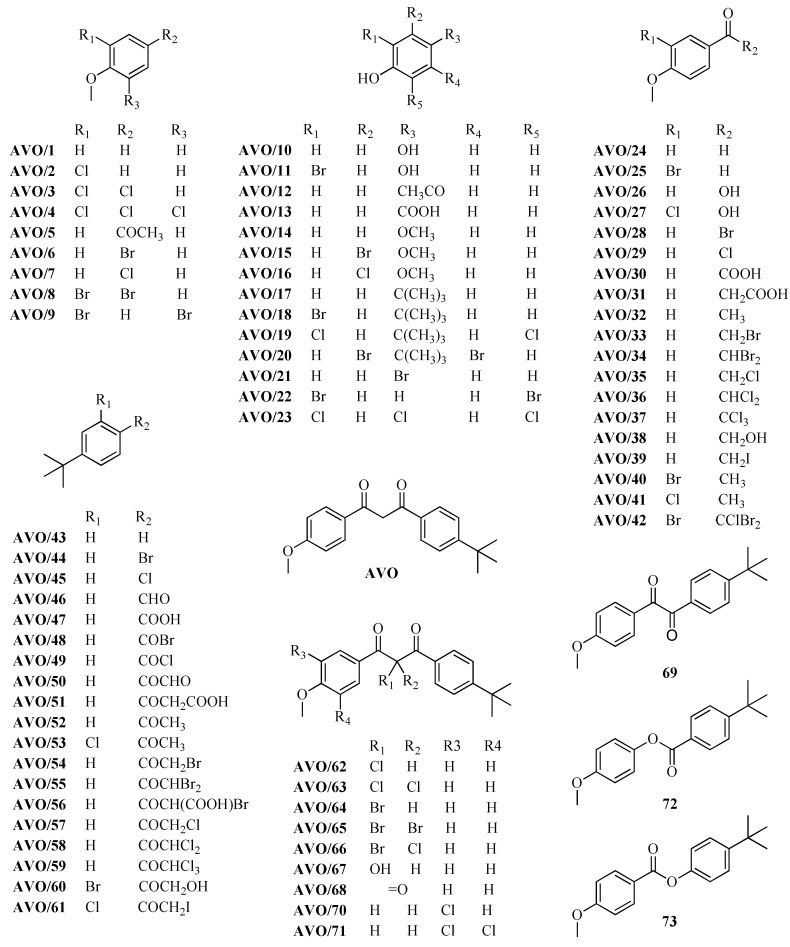
Disinfection by-products from **AVO**.

In order to fill the gaps in the literature, an assessment of the toxicity of these products on human health would be both beneficial and interesting. In one study, during the chlorination of **AVO** in reconstituted seawater (with a concentration of about 70 mg/L of bromide at pH 8.0, excluding the presence and possible role of the organic material normally present in solution), the presence of other brominated carbon derivatives was detected on the alpha carbon between the two carbonyl groups, namely the brominated products **AVO/65** and **AVO/66** as well as increasing quantities of bromoform (**4**, Scheme 2)—the ultimate product of oxidative degradation [38] (Scheme 6)—as a function of time. Among all solar filters, **AVO** has been studied in more detail; various groups have evaluated its reactivity in the chlorination reaction in distilled water [39,40,41,42,43], in water in the presence of dissolved organic material [39], in swimming pool water [44], in seawater, [40,45] and in the bromination reaction with KBrO in water [44].

In addition, one study evaluated its transformation due to the effect of irradiation with UV rays, during which it was stable at a maximum absorption at 254 nm [42]. On the contrary, it was more reactive when irradiated in a solution of water and acetonitrile (1:1)—where the formation of products in increasing quantities was observed as a function of the irradiation time (from 1 to 4 h) and generally identified by GC-MS analysis—or when irradiation and chlorination were combined [35]. It was possible to identify at least 74 products (**AVO/1-73** (Scheme 6) and compound **4** (Scheme 2)) by isolating them individually or identifying them via a comparison with reference compounds obtained by analogous mechanisms and with a reactivity similar to those proposed for the **BP-4** product. A series of considerations can be made: (i) In solution, the chlorination of the pure product is very fast—certainly faster than the active ingredient in commercial products, even with the combined action of Cl_2_/UV-B and UV-A rays (with degradation not exceeding 15%) and a large number of derivatives of acetophenone, benzoic acid, phenols, and benzaldehyde are obtained; (ii) The overall picture tends to become even more complicated in the presence of bromine, which results in the generation of different products.

Bromine, like chlorine, is particularly suitable for disinfecting swimming pool water. Bromine attacks bacteria, viruses, and fungi and, consequently, eliminates organic water pollution by oxidation, neutralizing polluting factors [37]. As in the case of chlorination, with bromination the reaction seems to start with the addition of bromine to the double bond of the enol form of AVO (**AVO/62-67**), which is then followed by hydrolysis and the formation of the corresponding tricarbonyl (**AVO/69**). Subsequently, a series of derivatives are obtained, which can then undergo chlorination on the aromatic ring, activated by the presence of an electron donor group, such as a methoxyl group. The reaction can be catalyzed up to 10 times by the addition of copper salts. Furthermore, in this case, there is a considerable increase in bromoform (**4**), the final product of the haloform reaction, with long treatments (at least 24 h). Certainly, some of the previously discussed by-products could also be derived from different molecules, for example, from those of anthropogenic origin; however, this would aggravate the general picture even more. The addition of KI apparently does not lead to the formation of iodinated derivatives but rather to a simple increase in chlorinated products, compared to the quantities obtained in the absence of KI itself [36]. Ecotoxicity tests on the unicellular bacterium *Vibrio fischeri* showed that, generally, all the products identified, individually considered or in a mixture, were more toxic than the pure molecule [37].

Table 3 shows the products obtained during the chlorination reaction in pure water or in distilled water in the presence of ions (Br^−^, I^−^, Cu^2+^ and Fe^3+^) as well as in swimming pool water (SPW), seawater (SW), dissolved organic material (DOM), and in the presence of UV radiation.

Table 4 shows the products obtained during the irradiation reaction with UV light or by reaction with NaBrO.

### 2.3. UVB Chemical Filters: Derivatives of p-Aminobenzoic Acid

**Scheme 7:**
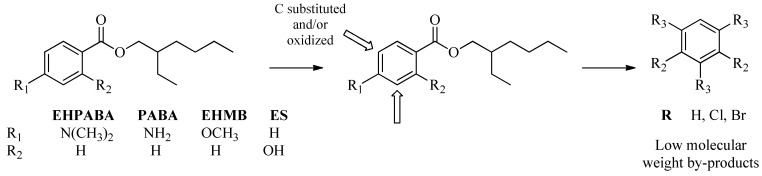
Chemical filters derived from *p*-aminobenzoic acid and possible reactive sites.

In the literature, there is less data on the chlorination reactions of *p*-aminobenzoic acid derivatives; however, all studies agree that aromatic proton substitution reactions occur at the positions most activated by the electron donor groups of the substituents already present on the ring (Scheme 7).

#### 2.3.1. 2-Ethylhexyl 4-(Dimethylamino)benzoate (**EHPABA**)

**EHPABA**, both the pure product and that contained in a commercial solar filter, at concentrations between 10 and 50 ng/mL when treated with sodium hypochlorite (0.1–3.0 μg/mL) has been shown to degrade by no more than 20% at pH = 8.2 after 10 min; at a lower pH and higher oxidant concentration, it degrades up to 80%, with pseudo-first-order degradation kinetics [25].

**Figure 4:**
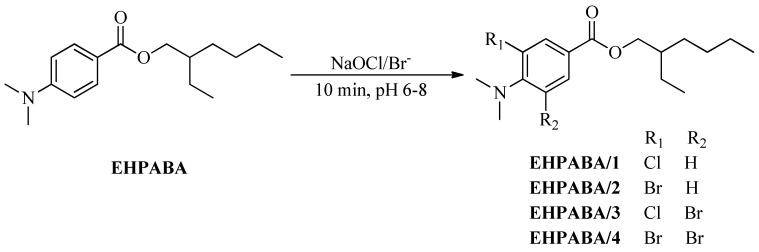
Disinfection by-products from **EHPABA** chlorination.

It exhibits a t_1/2_ of about 27 min in pure water and in the absence of bromide ions, which is reduced to less than 5 min in drinking water and in the presence of bromides. In one study, the behavior of **EHPABA** in a chlorination process was investigated with increasing concentrations of chlorine and in the presence or absence of bromine. During chlorination, the half-life times of the starting product showed a decrease that was inversely proportional to the concentration of bromine. In addition, four products were isolated and identified: two monohalogenated products, **EHPABA/1** and **EHPABA/2**, found in quantities 100 times higher than the two dihalogenated products, **EHPABA/3** and **EHPABA/4** (Figure 4).

The quantity of the two dihalogenated products increased slowly while the concentration of the monohalogenated products remained almost constant, indicating that the dihalogenated compounds represent a small percentage of halogenated compounds [40].

It should be noted that **EHPABA** does not appear to be genotoxic but the mixture obtained by bromination is [9]. It should be emphasized, however, that the use of increasing amounts of bromine does not lead to an increase in the toxicity of the mixture obtained.

#### 2.3.2. 2-Ethylhexyl 4-Methoxybenzoate (**EHMB**)

**Figure 5:**
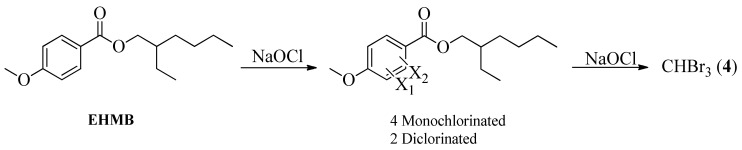
Disinfection by-products from **EHMB** chlorination.

In the case of **EHMB**, treatment with sodium hypochlorite at low concentrations first led to the formation of four monochloro derivatives and two dichloro derivatives (Figure 5). Compared to **EHPABA**, the products were more stable at an acidic pH (5 instead of 9). The mixtures obtained showed a weak mutagenic activity on *Salmonella typhimurium* [9,33].

### 2.4. UVB Chemical Filters: Derivatives of Salicylic Acid

#### 2-Ethylhexyl Salicylate (**ES**)

For **ES**, the same considerations made for **OC** apply: the product appears to be poorly reactive and is recovered unchanged at concentrations between 10 and 50 ng/mL after treatment with sodium hypochlorite at concentrations between 0.1 and 3.0 μg/mL, at a pH between 6 and 8, and at a temperature of about 20 °C [32].

**Figure 6:**
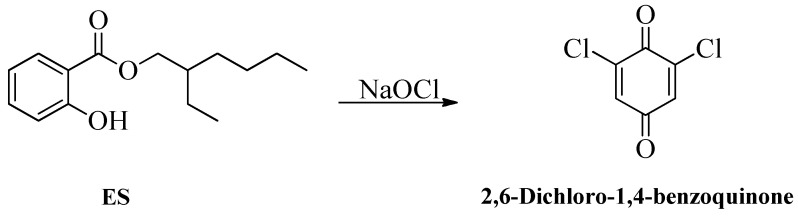
Disinfection by-products from **ES** chlorination.

It was demonstrated by Kosaka et al. [47] that a chlorination reaction with about 1–2 mg/L of Cl_2_ led to traces of 2,6-dichloro-1,4-benzoquinone; however, the authors did not specify the mechanism of its formation (Figure 6).

### 2.5. UVB Chemical Filters: Derivatives of Cinnamic Acid

**Figure 7:**
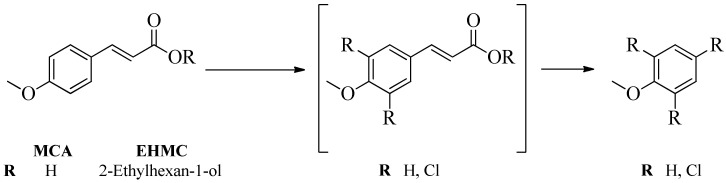
Chemical filters derived from cinnamic acid and possible reactive sites.

There is little data on the chlorination reaction of methoxycinnamic acid (**MCA**) and 2-ethylhexyl-4-methoxycinnamate (**EHMC**); this is likely due to the difficulty of detecting and isolating compounds with a single aromatic ring and a low molecular weight. In general, as is expected, the possible chlorination of the aromatic ring has been noted in the positions adjacent to those bearing the methoxyl group and the saponification of the ester bond (Figure 7). The ultimate degradation products are C_6_C_0_ skeletal aromatic compounds or their corresponding quinones. Below, we report on an analysis of oxidation products obtained from two derivatives of cinnamic acid.

#### 2.5.1. 4-Methoxycinnamic Acid (**MCA**)

**Figure 8:**
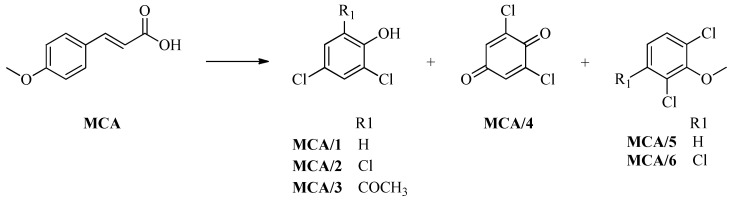
Disinfection by-products from **MCA** chlorination.

A substantially similar picture was also found for the reaction of 4-methoxycinnamic acid (**MCA**) with HClO, where the formation of 2,4-dichlorophenol (**MA/1**), 2,4,6-trichlorophenol (**MCA/2**), 3,5-dichloro-2-hydroxyacetophenone (**MCA/3**), 2,6-dichloro-1,4-benzoquinone (**MCA/4**), 1,3-dichloro-2-methoxybenzene (**MCA/5**), and 1,2,4-trichloro-3-methoxybenzene (**MCA/6**) was observed (Figure 8) [46].

The isolated and identified products are in accordance with the density functional theory (DFT), which indicates the higher reactivity of the double bond to electrophilic attack compared to the adjacent benzene aromatic ring, which subsequently undergoes the addition of HClO.

#### 2.5.2. 2-Ethylhexyl Methoxycinnamate (**EHMC**)

**Figure 9:**
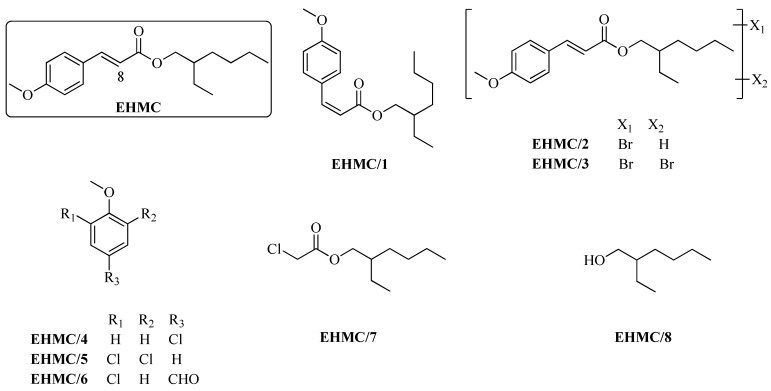
Disinfection by-products from **EHMC** chlorination.

The reaction of 2-ethylhexyl-4-methoxycinnamate (**EHMC**) with HOCl was shown to give rise to the formation of seven products, among which were **Z-EHMC** (**EHMC/1**), a geometric isomer of the starting product with a *Z* configuration at the double bond conjugated with the carbonyl group; two unidentified monochlorinated byproducts (**EHMC/2**,**3**); three anisole derivatives, namely 1-chloro-4-methoxybenzene (**EHMC/4**), 1,3-dichloro-2-methoxybenzene (**EHMC/5**). and 3-chloro-4-methoxybenzaldehyde (**EHMC/6**); and 2-ethylhexyl chloroacetate (**EHMC/7**; Figure 9). The same solutions treated with NaClO were irradiated between 200 and 600 nm for 3 h, and the geometric isomer **EHMC/1** and 2-ethylhexylalcohol (**EHMC/8**) were exclusively obtained, while chlorination and concomitant irradiation led to the formation of the products **EHMC/1**,**2**,**3** and **8**. It is interesting to note that the density functional theory indicates that the addition of HClO on the double bond conjugated to the carbonyl group is favored over the chlorination of the aromatic ring (by virtue of an accumulation of negative charge on the C-8 atom compared to the phenyl ring, as described by Gackowska et al. [46]).

## 3. Conclusions

This work provides an overview of the most widely used and well-known organic sunscreens from a commercial point of view and their environmental fate during disinfection processes. Some, or possibly all, of these products pose a potential environmental risk. Some studies have described the long-term damage to the aquatic systems where their concentrations were higher. They have been found both in coastal and seaside areas and even in swimming pool waters, and it has been shown that their environmental fate can depend on various factors, such as the pH of the water, the presence of organic material, metal ions and light, and, above all, on the chemical agents used in the disinfection of the water itself (primarily sodium hypochlorite). So, for each sunscreen, the possible environmental fate is described when the waters in which they are present are subjected to the disinfection processes necessary to lower the bacterial load. These processes are capable of generating dozens of products with a lower molecular weight and generally of an aromatic nature (benzaldehydes, acetophenones, phenols, anisoles, etc.), of which only some have received an exact structural definition and evaluation of their precise toxicological profile. If the efficiency of sunscreens is currently considered to be high, the same cannot be said for the methods used to eliminate and prevent their presence in the environment, and this should suggest the necessity for more efficient removal methods or the redesign of sunscreens not only for the purposes for which they are used.

## Figures and Tables

**Table 1:** Abbreviation, name, commercial name, and CAS number of sunscreens.

Abbreviation	Name	Commercial Names	CAS Number
**BP-3**; OXY	2-Benzoyl-5-methoxyphenol	Benzophenone-3; Eusolex 4360; Escalol 567; Uvinul 3040	131-57-7
**BP-4**	5-Benzoyl-4-hydroxy-2-methoxybenzenesulfonic acid	Benzophenone-4; Escalol 577; Uvasorb S 5; Uvinul D 5030	4065-45-6
**BP-8**; DIOXY; DMB	4-Methoxy-2,2′-dihydroxy-benzophenone	Benzophenone-8; Dioxibenzone; Evesorb 52; Cyasorb UV 24	131-53-3
**DHHB**	Hexyl 2-(4-diethylamino-2-hydroxybenzoyl)benzoate	Uvinocamphor l Plus A; Uvinul A+	302776-68-7
OC	2-Ethylhexyl-2-cyano-3,3-diphenylacrylate	Octocrylene; Escalol 597; Uvinul 3039; Uvinul N 539	6197-30-4
**AVO**; BMDM	4-*tert*-Butyl-4′-methoxy-dibenzoylmethane	Avobenzone; Parsol 1789; Eusolex 9020	70356-09-1
**EHPABA**; EHDAB; ODPABA	2-Ethylhexyl 4-(dimethylamino)benzoate	Ethylhexyl dimethyl paba; Padimate O; Chemcure EHA; Escalol 507	21245-02-3
**PABA**	2-Ethylhexyl 4-aminobenzoate	Vitamin Bx	26218-04-2
**EHMB**	2-Ethylhexyl 4-methoxybenzoate	-	16451-27-7
**ES**	2-Ethylhexyl salicylate	Soct; Sunobe OS	118-60-5
**MCA**	4-Methoxycinnamic acid	NSC 5303	830-09-1
**EHMC**	2-Ethylhexyl methoycinnamate	Escalol 557; Parsol MCX; Uvinul 3088; Octinoxate	5466-77-3

**Table 2:** Some chemical and physical characteristics of the solar filters considered.

Abbreviation	MS	Formula	ε (L mol^−1^ cm^−1^)	Octanol–Water Partition Coefficient (Log K_ow)_	Solubility (g L^−1^ at 25 °C and pH 6–7)	Refs.
**BP-3**	228.24	C_14_H_12_O_3_	9300 at λ = 321 nm	3.79		[5,6]
**BP-4**	308.31	C_14_H_12_O_6_S	6700 at λ = 319 nm	6.3		[7]
**BP-8**	224.24	C_14_H_12_O_4_	9400 at λ = 326 nm	4.31		[6]
**DHHB**	397.51	C_24_H_31_NO_4_	35,900 at λ = 355 nm	6.93	9.5 × 10^−4^	[8,9]
**OC**	361.48	C_24_H_27_NO_2_	1300 at λ = 355 nm	6.88	3.81 × 10^−6^	[5,10,11]
**AVO**	310.39	C_20_H_22_O_3_	33,756 at λ = 360 nm	4.51		[5,12,13]
**EHPABA**	277.40	C_17_H_27_NO_2_	28,400 at λ = 316 nm	5.76	5.3 × 10^−6^	[5,6,10,14]
**PABA**	249.35	C_15_H_23_NO_2_	19,300 at λ = 282 nm	4.22		[15,16]
**EHMB**	290.40	C_18_H_26_O_3_	24,200 at λ = 312 nm	5.80	1.55 × 10^−4^	[5,6,10]
**ES**	250.33	C_15_H_22_O_3_	5200 at λ = 260 nm	5.97		[5,17]
**MCA**	178.18	C_10_H_10_O_3_	18,800 at λ = 329 nm			[18]
**EHMC**	290.40	C_18_H_26_O_3_	24,200 at λ = 312 nm	5.80		[6,14]

**Table 3:** Products obtained by hypochlorination in the presence of light and ions and in different aqueous matrices.

AVO/	Common Name	NaOCl									Refs.
		-	Br^−^	I^−^	Cu^2+^	Fe^3+^	SPW	SW	DOM	hν	
**1**	Anisole							✓			[45]
**2**	Anisole, 2-chloro									✓	[42]
**3**	Anisole, 2,4-dichloro	✓			✓						[42,43]
**4**	Anisole, 2,4,6-trichloro	✓									[42]
**5**	Anisole, 4-acetyl	✓	✓					✓			[43,45]
**6**	Anisole, 4-bromo	✓	✓					✓			[43,44,45]
**7**	Anisole, 4-chloro	✓	✓	✓	✓			✓			[42,43,45]
**8**	Anisole, 2,4-dibromo		✓					✓			[43,45]
**10**	Hydroquinone							✓			[44,45]
**12**	Acetophenone, 4-hydroxy							✓			[45]
**13**	Benzoic acid, 4-hydroxy							✓			[45]
**14**	Phenol, 4-methoxy	✓	✓	✓	✓			✓			[42,43,44,45]
**15**	Phenol, 3-bromo-4-methoxy							✓			[44,45]
**16**	Phenol, 3-chloro-4-methoxy	✓									[42]
**17**	Phenol, 4-*tert*-butyl	✓	✓	✓	✓	✓	✓	✓			[42,43,44,45]
**18**	Phenol, 2-bromo-4-*tert*-butyl							✓			[45]
**20**	Phenol, 3,5-dibromo-4-*tert*-butyl	✓									[44]
**21**	Phenol, 4-bromo							✓			[44,45]
**24**	Benzaldehyde, 4-methoxy	✓	✓	✓	✓	✓	✓	✓		✓	[42,43,44,45]
**25**	Benzaldehyde, 3-bromo-4-methoxy							✓			[37,45]
**26**	Benzoic acid, 4-methoxy	✓	✓	✓	✓	✓		✓		✓	[42,43,44,45]
**27**	Benzoic acid, 3-chloro-4-methoxy	✓									[42]
**28**	Benzoyl bromide, 4-methoxy							✓			[45]
**29**	Benzoyl chloride, 4-methoxy	✓	✓	✓	✓	✓	✓	✓			[42,43,44,45]
**31**	Benzoyl acetic acid, 4-methoxy	✓	✓	✓	✓						[43,44]
**32**	Acetophenone, 4-methoxy		✓	✓	✓	✓	✓	✓			[43,44]
**33**	Acetophenone, α-bromo-4-methoxy	✓	✓					✓			[43,44,45]
**34**	Acetophenone, α,α-dibromo-4-methoxy							✓			[45]
**35**	Acetophenone, α-chloro-4-methoxy	✓	✓	✓	✓	✓					[41,42,43]
**36**	Acetophenone, α,α-dichloro-4-methoxy	✓	✓	✓	✓	✓				✓	[42,43]
**37**	Acetophenone, α,α,α-trichloro-4-methoxy	✓									[42]
**38**	Acetophenone, α-hydroxy-4-methoxy	✓	✓	✓	✓			✓			[42,45]
**39**	Acetophenone, α-iodo-4-methoxy	✓		✓							[42]
**40**	Acetophenone, 3-bromo-4-methoxy							✓			[44,45]
**41**	Acetophenone, 3-chloro-4-methoxy							✓			[45]
**42**	Acetophenone, α,α-dibromo, α-chloro-4-methoxy							✓			[45]
**43**	Benzene, *tert*-butyl							✓			[44,45]
**44**	Benzene, 1-bromo-4-*tert*-butyl							✓			[44,45]
**45**	Benzene, 1-chloro-4-*tert*-butyl									✓	[42]
**46**	Benzaldehyde, 4-*tert*-butyl	✓	✓	✓	✓	✓		✓		✓	[42,43,44,45]
**47**	Benzoic acid, 4-*tert*-butyl	✓	✓	✓	✓	✓	✓	✓		✓	[42,43,44,45]
**48**	Benzoyl bromide, 4-*tert*-butyl							✓			[45]
**49**	Benzoyl chloride, 4-*tert*-butyl	✓	✓	✓	✓	✓	✓	✓			[42,43,45]
**51**	Benzoyl acetic acid, 4-*tert*-butyl	✓	✓	✓	✓	✓	✓	✓			[43]
**52**	Acetophenone, 4-*tert*-butyl	✓	✓	✓	✓	✓	✓	✓			[43,44,45]
**53**	Acetophenone, 2-chloro-4-*tert*-butyl							✓		✓	[45]
**54**	Acetophenone, α-bromo-4-*tert*-butyl	✓	✓					✓			[43,44,45]
**55**	Acetophenone, α,α-dibromo-4-*tert*-butyl							✓			[45]
**56**	Acetophenone, α-bromo,α- carboxy-4-*tert*-butyl							✓			[45]
**57**	Acetophenone, α-chloro-4-*tert*-butyl	✓	✓	✓	✓	✓		✓			[42,43,45]
**58**	Acetophenone, α,α-dichloro-4-*tert*-butyl	✓	✓	✓	✓	✓	✓	✓		✓	[42,43,45]
**59**	Acetophenone, α,α,α-trichloro-4-*tert*-butyl	✓								✓	[42]
**60**	Acetophenone, α-hydroxy-2-bromo -4-*tert*-butyl	✓	✓	✓	✓			✓			[43]
**61**	Acetophenone, α-iodo-2-chloro-4-*tert*-butyl	✓		✓							[43]
**62**	Chloroavobenzone	✓	✓	✓	✓	✓		✓		✓	[42,43,44,45]
**63**	Dichloroavobenzone	✓	✓	✓	✓	✓		✓		✓	[42,43,44,45]
**64**	Bromoavobenzone	✓	✓					✓			[40,43,44,45]
**65**	Dibromoavobenzone	✓	✓					✓			[40,43]
**66**	Bromo-chloroavobenzone	✓	✓								[43]
**68**	1-(4-*tert*-Butyl)phenyl-3-(4-methoxy)- Phenyl propantrione-1,2,3	✓	✓	✓	✓	✓		✓			[43,44,45]
**69**	1-(4-*tert*-Butyl)phenyl-2-(4-methoxy)- phenylethandione-1,2	✓	✓	✓	✓	✓		✓			[43,44,45]
**70**	4-*tert*-Butyl-3′-chloro-4′- methoxydibenzoylmethane								✓		[39]
**71**	4-*tert*-Butyl-3′,5′-dichloro-4′- methoxydibenzoylmethane								✓		[39]
	Bromoform (**4**)							✓			[40]

**Table 4:** Products obtained by UV irradiation and during a hypobromination process.

No.	Common Name	Treatment		Refs.
		hν	NaOBr	
**1**	Anisole		✓	[45]
**9**	Anisole, 2,6-dibromo		✓	[44]
**10**	Hydroquinone		✓	[44,45]
**11**	Hydroquinone, 2-bromo		✓	[44]
**14**	Phenol, 4-methoxy	✓	✓	[42,43,44,45]
**15**	Phenol, 3-bromo-4-methoxy		✓	[44,45]
**17**	Phenol, 4-*tert*-butyl	✓	✓	[42,43,44,45]
**19**	Phenol, 2,6-dichloro-4-*tert*-butyl		✓	[42]
**22**	Phenol, 2,6-dibromo		✓	[44]
**23**	Phenol, 2,4,6-trichloro		✓	[42]
**24**	Benzaldehyde, 4-methoxy	✓	✓	[42,43,44,45]
**25**	Benzaldehyde, 3-bromo-4-methoxy		✓	[44,45]
**26**	Benzoic acid, 4-methoxy	✓	✓	[42,43,44,45]
**30**	Benzoyl formic acid, 4-methoxy		✓	[44]
**31**	Benzoyl acetic acid, 4-methoxy		✓	[43,44]
**32**	Acetophenone, 4-methoxy		✓	[44]
**33**	Acetophenone, α-bromo-4-methoxy		✓	[43,44,45]
**40**	Acetophenone, 3-bromo-4-methoxy		✓	[44,45]
**43**	Benzene, *tert*-butyl		✓	[44,45]
**44**	Benzene, 1-bromo-4-*tert*-butyl		✓	[44,45]
**46**	Benzaldehyde, 4-*tert*-butyl	✓	✓	[42,43,44,45]
**47**	Benzoic acid, 4-*tert*-butyl	✓	✓	[42,43,44,45]
**50**	Benzoyl formaldehyde, 4-*tert*-butyl		✓	[44]
**52**	Acetophenone, 4-*tert*-butyl		✓	[43,44,45]
**53**	Acetophenone, 2-chloro-4-*tert*-butyl	✓		[45]
**54**	Acetophenone, α-bromo-4-*tert*-butyl		✓	[43,44,45]
**64**	Bromoavobenzone		✓	[40,43,44,45]
**67**	Hydroxyavobenzone		✓	[44]
**68**	ArCOCOCOAr’		✓	[43,44,45]
**69**	ArCOCOAr’		✓	[43,44,45,46]
**72**	AVO-8		✓	[44]
**73**	AVO-9		✓	[44]
